# How to assess refractive error in adults with additional or complex needs

**Published:** 2024-05-15

**Authors:** Michelle Hennelly, Irene Ctori, Poonam Shah, David Lewis

**Affiliations:** 1 Associate Professor: City, University of London, UK.; 2 Professor of Optometry: City, University of London, UK.; 3 Inclusive Eye Health Advisor: CBM Global Disability Inclusion, London, UK.; 4 Former CBM Focal Point for Inclusion in Eye Health, Melbourne, Australia.

**Being flexible, calm, and sensitive to patients’ needs and abilities is key to offering a patient-centred eye examination.**

**Figure:**
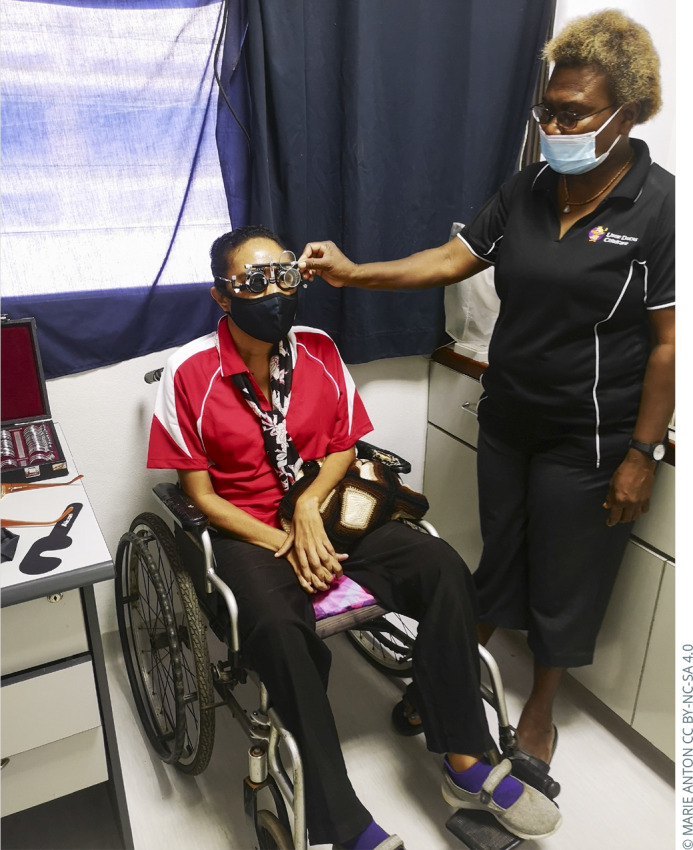
In low- and middle-income countries, 1 in 6 people have a significant disability. papua new guinea

A ‘person-centred’ approach to refractive error assessment shifts the focus from treating just a refractive error or medical condition, to treating the patient as a whole person. Every person is different, so we, as practitioners, need to tailor our approach to meet the individual needs of each patient.^1^

In low- and middle-income countries, more than 1 in every 8 people have a significant disability.^2^ In this article, we show how you can adapt your refractive error assessment process for adults with a range of sensory, physical, or cognitive differences (alone or in combination), which may not be immediately obvious and which the patient may not tell you about.

## Types of challenges

Patients can present with a range of challenges.

For example, challenges such as reduced mobility, abnormal head posture, motor coordination issues, and/or difficulties in maintaining a fixed gaze in a certain direction may make it difficult for the patient to see the eye chart.

Being deaf or hard of hearing, or having cognitive impairment, anxiety, or attention difficulties may make it difficult for the patient to understand and respond to questions, or to follow instructions.

## 1. Before the examination begins

- Ensure that the examination room is fully accessible to people who use a wheelchair.
- Observe the patient entering the room. Greet them and invite them to sit down. This is an opportunity to objectively assess the patient's physical abilities and restrictions, as well as their ability to understand and follow instructions.
- Allow the patient some time to familiarise themselves with the room and the equipment.
- When the patient is settled, introduce yourself and talk to the patient in a friendly way.
- Ask a few introductory questions, such as their name, how they are, and whether they are comfortable.
- Ask the patient if they have any communication preferences. Alternatively, if you notice that they have difficulties understanding you (e.g., due to being deaf or hard of hearing, or having cognitive difficulties) ask how you can make things easier for them (see panel)
- If the patient's support person is acting as an interpreter, continue to speak to the patient directly and maintain appropriate eye contact – **do not address the support person instead of the patient**.

**How to support communication and interaction with the patient**

Understanding how best to communicate with your patient is vital. Ask the patient if they have any communication preferences, then allow enough time for them to answer.

When your patient is deaf, hard of hearing, non-verbal, is from a different language group, or has other communication challenges, it is important to speak clearly and slowly. If patients rely on lip reading, remove your face mask (if safe to do so), and ensure that the patient can clearly see your face when you are speaking to them. Avoid making the room too dark, and don't position yourself in front of a bright light or bright window.

If patients have cognitive difficulties, speak slowly and use short, simple sentences. You can also use gestures or pictures to explain procedures and choices, if needed.

Always speak with the patient directly, even if they have an interpreter (e.g. a sign language interpreter). Never assume that the patient cannot understand you, and don't talk about the patient to the interpreter or support person, as if the patient isn't there.

## 2. Taking a history

- Ideally, you will have received information about the patient ahead of the assessment appointment. Some organisations request that patients or their support persons complete a form in the weeks leading up to the appointment, such as this one by the charity SeeAbility: www.seeability.org/resources/about-me-and-my-eyes. If no information is available, and you have time, it may be worth contacting the patient or support person by phone before the appointment to get a brief history.
- Ask about their current prescription (if any) and whether they are able to function as they would like to, in terms of both distance and near visual tasks. Do they have any other visual symptoms?
- Ensure that patients have enough time to answer your questions before moving them on to the next question. It is important that the patient feels that you understand their needs and that you want to help them.
- A useful general question with which to end history taking is, ‘Is there anything else you want to tell me?’

## 3. The examination

Being patient and flexible throughout the examination will help you to adapt your approach based on the patient's responses and needs. Remember to allow enough time for patients to follow instructions and answer questions.

For ease of examination, specific equipment and techniques may be needed.

Before **testing visual acuity**, find out whether patients are familiar with the alphabet; they may be more comfortable using Lea or Kay symbols or a tumbling E chart; matching cards can also be helpful. **Note:** Measuring visual acuity might not be possible or vital, so this can be elicited by asking what sort of tasks the patient does and what they can normally see. Think about what you are trying to achieve for the patient.

In terms of the **objective refractive routine**, retinoscopy is a vital skill and may be the only way to assess refraction for patients with significant physical or intellectual challenges. Cycloplegia may be recommended in young adults – see the article in this issue. If a dark room is anxiety-provoking for the patient, it is fine to keep the light on. Using loose trial lenses or a lens rack can also help to speed up the assessment. Obtaining an accurate result from this part of the routine will reduce the time spent on the subjective refraction, which will reduce the patient's fatigue.

You can simplify the **subjective refraction routine** by using larger differentials in power (e.g. plus or minus 1:00 D) or fewer lens choices; this can help to make the subjective examination more meaningful. Where possible, ask questions that are easy to understand. For example, ask if the lenses reduce blur, or help the patient to see smaller letters. Moving the chart or patient closer may be helpful, particularly in cases of abnormal head posture and those with visual impairment. A 3-metre test chart is useful, and using a logMAR chart will help you to account for different working distances. When assessing astigmatism, a 1:00 DC Jackson cross cylinder (used with an appropriate visual target), with the cylinder moved in 20-degree steps, will help patients with visual impairment discriminate differences more easily.

People with additional needs are more likely to need help with near vision. When assessing near vision in someone who is unable to communicate, **dynamic retinoscopy** can be very useful.^3^ It is important that any significant distance refractive error is corrected first, and that dynamic retinoscopy is done without cycloplegia.

If a patient can communicate the clarity of their near vision, then you can carry out a **subjective near vision assessment**. You can refine in 0.5 D or 1.00 D steps to elicit the most appropriate add at the preferred working distance. Remember that the level of clarity needed is determined by the near vision tasks a patient performs. Perfect near vision is not always needed.

**Tips for reducing patients’ anxiety**

- Allow enough time for the examination so that you are not rushed. If you are calm, the patient is more likely to feel calm.
- It may be helpful to modify the testing environment to suit the patient's needs. For example, you can change the lighting (not too bright, and not too dark), reduce distractions, and plan or suggest breaks from testing.
- Explain each step of the examination process as you perform them, in simple, non-technical language – this helps to build trust. You can do this even if you’re not sure how much the patient understands.
- If the patient has a cognitive impairment, a useful way to reduce their anxiety can be to carry out an examination task or test on their support person first; this helps them to feel more familiar with the situation and understand what is expected.

If the patient is of presbyopic age (usually over the age of 40), their age can be used to estimate the likely starting point for the level of add needed.^4^

In patients with **low vision**, a high reading add of +4.00 D or more can be used; however, the shorter working distance needs to be emphasised. Base-in prisms need to be considered to aid convergence for higher adds. A low vision aid may be more appropriate, this might require referral to a low vision service.

## After the examination

It is important to involve the patient in the decision-making process following the findings of the refractive examination. For example, if they require both near and distance vision correction, would they prefer to have different spectacles for each type of task, or would bifocal/progressive lenses work better for them? See the article on patient education in this issue for more information.

Respect patients’ preferences and consider their capabilities when suggesting solutions. If needed, additional recommendations around lighting, colour, and contrast may also be helpful to enable patients to make the most of their sight in different environments.

It may be useful to write down your findings to help the patient and/or support person remember your recommendations; for example, detailing which spectacles or low vision aid is used for each visual task, while also checking the availability and affordability of each device, and explaining where to get it and what funding may be available to help with the costs, if needed.

In order to plan follow-up, it is very important to understand the patient's circumstances in coming for their eye examination. For example: Have they come from a long distance? Has their support worker needed to give up a day's paid work in order to accompany them? Has the visit created a large financial burden? Can the patient be seen locally, or is a mobile eye team visit planned to the patient's area?

Provide clear instructions for any follow-up care, together with contact details in case there are any further questions; this will help to reduce anxiety and make it possible to address any issues that may come up in future.
